# Establishment of a risk prediction model for olfactory disorders in patients with transnasal pituitary tumors by machine learning

**DOI:** 10.1038/s41598-024-62963-7

**Published:** 2024-05-31

**Authors:** Min Chen, Yuxin Li, Sumei Zhou, Linbo Zou, Lei Yu, Tianfang Deng, Xian Rong, Shirong Shao, Jijun Wu

**Affiliations:** 1 Department of Neurosurgery, Deyang People’s Hospital, Deyang, 618000 China; 2 School of Nursing, North Sichuan Medical College, Nanchong, 637000 China; 3 Department of Nursing, Deyang People’s Hospital, Deyang, 618000 Sichuan China; 4 Institute of Complex Systems, Shanxi University, Taiyuan, 030001 China; 5 Sichuan Nursing Vocational College, Chengdu, 610110 China

**Keywords:** Pituitary tumor, Olfactory impairment, Transnasal pterygoid region, Machine learning, Predictive models, Neuroscience, Neurology

## Abstract

To construct a prediction model of olfactory dysfunction after transnasal sellar pituitary tumor resection based on machine learning algorithms. A cross-sectional study was conducted. From January to December 2022, 158 patients underwent transnasal sellar pituitary tumor resection in three tertiary hospitals in Sichuan Province were selected as the research objects. The olfactory status was evaluated one week after surgery. They were randomly divided into a training set and a test set according to the ratio of 8:2. The training set was used to construct the prediction model, and the test set was used to evaluate the effect of the model. Based on different machine learning algorithms, BP neural network, logistic regression, decision tree, support vector machine, random forest, LightGBM, XGBoost, and AdaBoost were established to construct olfactory dysfunction risk prediction models. The accuracy, precision, recall, F1 score, and area under the ROC curve (AUC) were used to evaluate the model's prediction performance, the optimal prediction model algorithm was selected, and the model was verified in the test set of patients. Of the 158 patients, 116 (73.42%) had postoperative olfactory dysfunction. After missing value processing and feature screening, an essential order of influencing factors of olfactory dysfunction was obtained. Among them, the duration of operation, gender, type of pituitary tumor, pituitary tumor apoplexy, nasal adhesion, age, cerebrospinal fluid leakage, blood scar formation, and smoking history became the risk factors of olfactory dysfunction, which were the key indicators of the construction of the model. Among them, the random forest model had the highest AUC of 0.846, and the accuracy, precision, recall, and F1 score were 0.750, 0.870, 0.947, and 0.833, respectively. Compared with the BP neural network, logistic regression, decision tree, support vector machine, LightGBM, XGBoost, and AdaBoost, the random forest model has more advantages in predicting olfactory dysfunction in patients after transnasal sellar pituitary tumor resection, which is helpful for early identification and intervention of high-risk clinical population, and has good clinical application prospects.

## Introduction

Pituitary tumors, also known as pituitary neuroendocrine tumors, are a group of heterogeneous lesions of the central nervous system and are common benign tumors that account for approximately 10–20% of intracranial tumors, making them the second largest intracranial tumors in humans, after meningiomas^1,2^. With the advancement of medical technology, especially optical and electrodescent technology, pituitary tumor resection via endoscopic transnasal pterygoid region has become the treatment of choice^3,4^. Despite the advantages of endoscopic surgery, such as less trauma and faster recovery, 10.5–44% of patients still experience olfactory impairment due to trauma to the nasal mucosa, septum, and turbinates during this procedure due to the unavoidable damage to the nasal structures^5,6^. Olfactory function is essential for daily life and is related to the perception of food flavors, the detection of potentially hazardous substances, and the maintenance of personal hygiene^7^. Despite the increasing sophistication of the surgical technique of transsphenoidal pituitary tumor removal postoperative nasal complications, the clinical assessment of olfactory dysfunction still relies heavily on olfactory function tests such as the UPSIT (University of Pennsylvania Smell Identification Test), and in particular, the assessment of intelligent prediction of olfactory dysfunction that has received relatively little attention in clinical research^8^. In recent years, machine learning algorithms have been increasingly used in the medical field, especially in the risk prediction of diseases, demonstrating advantages unmatched by traditional statistical methods, such as in the accuracy of model construction and ease of operation^9^. However, there are still fewer studies on risk prediction models for postoperative olfactory impairment in pituitary tumors. Given this, this study utilized real-world clinical data to process in-depth data and analyze patients' clinical information after transsphenoidal pituitary tumor resection. We used various machine learning algorithms to construct risk prediction models for olfactory impairment, such as BP neural network model, logistic regression model (LR), decision tree model(DT), support vector machine model (SVM), random forest model (RF), LightGBM model, XGBoost model, and AdaBoost model. By comparing and screening different models, this study aims to find the optimal prediction model to improve the prediction accuracy of olfactory dysfunction after pituitary tumor surgery, which provides new insights into understanding the mechanism of olfactory dysfunction as well as possible preventive measures, provides more effective clinical decision support, and improves the quality of life of patients and their overall recovery after surgery.

## Objects and methods

### Subjects

Patients with pituitary tumors in the neurosurgery departments of three tertiary general hospitals in Sichuan Province (West China Hospital of Sichuan University, People's Hospital of Deyang City, and Affiliated Hospital of Chengdu University) from January to December 2022 were used as the study subjects by using a cross-sectional study design. Inclusion criteria: (1) Preoperative cranial imaging (CT or MRI) confirmed the diagnosis of saddle region occupancy; (2) Postoperative pathology showed pituitary adenoma; (3) First time to perform pituitary tumor surgery, and the surgical procedure was selected as neuro endoscopic transnasal butterfly approach pituitary tumor resection; (4) Age greater than 18 years old; (5) Agreed to participate in the study and signed an informed consent form. Exclusion criteria: (1) Cases with a history of head trauma, nasal surgery, transnasal approach surgery and radiation therapy; (2) Cases with history of hypersensitivity during olfactory test; (3) Cases with olfactory sulcus meningiomas, saddle-node meningiomas, and other causes of subjective olfactory impairment; (4) Cases who did not cooperate with the study and had no effective follow-up; (5) Incomplete information on key predictive factors and predicted outcome status. The study design was by the Declaration of Helsinki of the World Medical Association and was reviewed by the Medical Ethics Membership of Deyang People's Hospital before the study began (Ethics Review No. 2021-04-059-K01).

## Methods

### Study variables

The outcome index is based on whether or not patients develop olfactory impairment after surgery. Predictive variables of olfactory impairment after transnasal pyriform saddle region pituitary tumor resection were set up by combining literature reports and clinical experience, which mainly included: (1) Demographic and sociological characteristics: age, gender, smoking history, drinking history, allergy history; (2) Disease-related factors: lesion location (In-saddle, in-saddle-on-saddle, in-saddle-on-saddle-by-saddle),size of pituitary tumors (microadenomas: diameter < 10 mm, macroadenomas: diameter ≥ 10 mm), pituitary tumor type (prolactin adenoma (PRL), growth hormone tumor (GH), adrenocorticotropic hormone-stimulating adenoma (ACTH), thyrotropin-stimulating hormone tumor (TSH), luteinizing hormone/follicle-stimulating hormone tumor (LH/FSH), non-functioning pituitary tumor), stroke of pituitary tumor (yes, no); length of the operation (≤ 2 h, > 2 h) (3) Postoperative nasal condition: formation of blood crust ( yes, no), mucosal erosion (yes, no), nasal adhesions (yes, no), sinus effusion (yes, no), cerebrospinal fluid rhinorrhea (yes, no) (4) Underlying diseases: hypertension, diabetes mellitus, coronary artery disease, chronic renal disease, chronic obstructive pulmonary disease, etc., which were dichotomously categorized by combining one or more of the underlying diseases (yes, no) were transformed and analyzed.

### Determination of olfactory impairment

The five-flavor olfactory test was used to determine the patients' sense of smell^5^. This study used the olfactory braid assessment solution (00016382280) produced by Daiichi Pharmaceuticals, Japan, to conduct the olfactory test by the “5-2 method”. The five odors were A (β-phenyl ethanol, floral odor, − 4.0), B (methylcyclopentenolone, burnt odor, − 4.5), C (isovaleric acid, sweaty odor, − 5.0) D (γ-undecanocaproic (alkyl) acid lactone, rotten fruit odor, − 4.5) E (β-methylindole, fecal odor, − 5.0). TEST METHOD: (1) The operator records the numbers 1–5 on the odor paper. The two sheets' front ends (roughly 1 cm apart) were then dipped into a baseline odor liquid. The other three sheets were dipped the same way into a control liquid and removed. (2) The subject sniffs each of the five pieces of paper (nose tip close, not touching) and writes the serial numbers of the two pieces of paper with an odor. (3) Repeat the same procedure for the other odors so that the test subjects can identify the types of odors carried by the odor papers. Test results: All correct answers are considered “normal,” and the results are “abnormal” if two pieces of paper with odors cannot be wholly identified as having one kind of odor or if a certain kind of odor cannot be identified.

### Questionnaire of olfactory disorders

A questionnaire of olfactory disorders-negative statements (QOD-NS) was used^10^. The Questionnaire contained 17 negative descriptions, using a 4-point scale, consisting of a score of 3 indicating that the situation in the description does not exist, a score of 0 indicating complete agreement, and a total score of 0–51. The closer the score is to the total score of 51, the more inclined one is to have an ordinary sense of smell, and on the contrary, the lower the score is, the more severe the olfactory disorder is.

### Data processing

#### Data description

In this study, information on pituitary tumor patients in three tertiary general hospitals in Sichuan Province (West China Hospital of Sichuan University, People's Hospital of Deyang City, and Affiliated Hospital of Chengdu University) was collected from January-December 2022. The dataset consisted of 158 samples, with 17 dimensions of the explanatory variables and one dimension of the explanatory variables, among which all 16 dimensions were subtypes except for age, which was a numerical variable.

#### Data analysis

In the data set of 158 cases, there were 83 male patients and 75 female patients; among male postoperative pituitary tumor patients, 61 had olfactory impairment, and 22 had no olfactory impairment. Among the female patients, the number of patients with and without olfactory impairment was 55 and 20, respectively. Among all 158 postoperative pituitary tumor patients, 116 (73.42%) had olfactory dysfunction, and 42 (26.58%) had no olfactory dysfunction. The distribution of gender and the number of patients with olfactory impairment is shown in Table 1.

**Table 1:** Distribution statistics of gender and number of people with olfactory impairment.

Sex	Yes	No	Total
Male	61	22	83
Female	55	20	75
Total	116	42	158

#### Data cleaning and processing of missing values

All variables were screened, data cleaning was performed, cases with missing information or missing values of more than 25% were deleted, and patients with more than 5% missing personal data cases were excluded. The rest of the missing were filled in according to multiple interpolations. Since there were no patients with less than 5% missing personal data of cases in this study, the raw data were processed directly.

The dimension of the original dataset is (158 × 20). The two features of serial number and name coupled in the dataset are eliminated. Then, the highly unbalanced pituitary tumor size feature is removed to obtain a dataset with the dimension of (158 × 17). We process the two features of age and lesion site: the decision tree split-box discretization method is used for the age feature, and the results of the split-box are shown in Table 2. The results are shown in Table 6. As can be seen from the table, there are eight age intervals after using the decision tree discretization, and about half of the number of people (70) are assigned to the interval (37, 56]. In comparison, the remaining 88 people are assigned to the other seven intervals with an approximately uniform distribution.

**Table 2:** Statistics on the number of persons by age box.

Age	Numbers
< = 37	12
(37,56]	70
(56,58]	15
(58,60]	12
(60,63]	10
(63,66]	14
(66,68]	12
> 68	13

**Figure 1:**
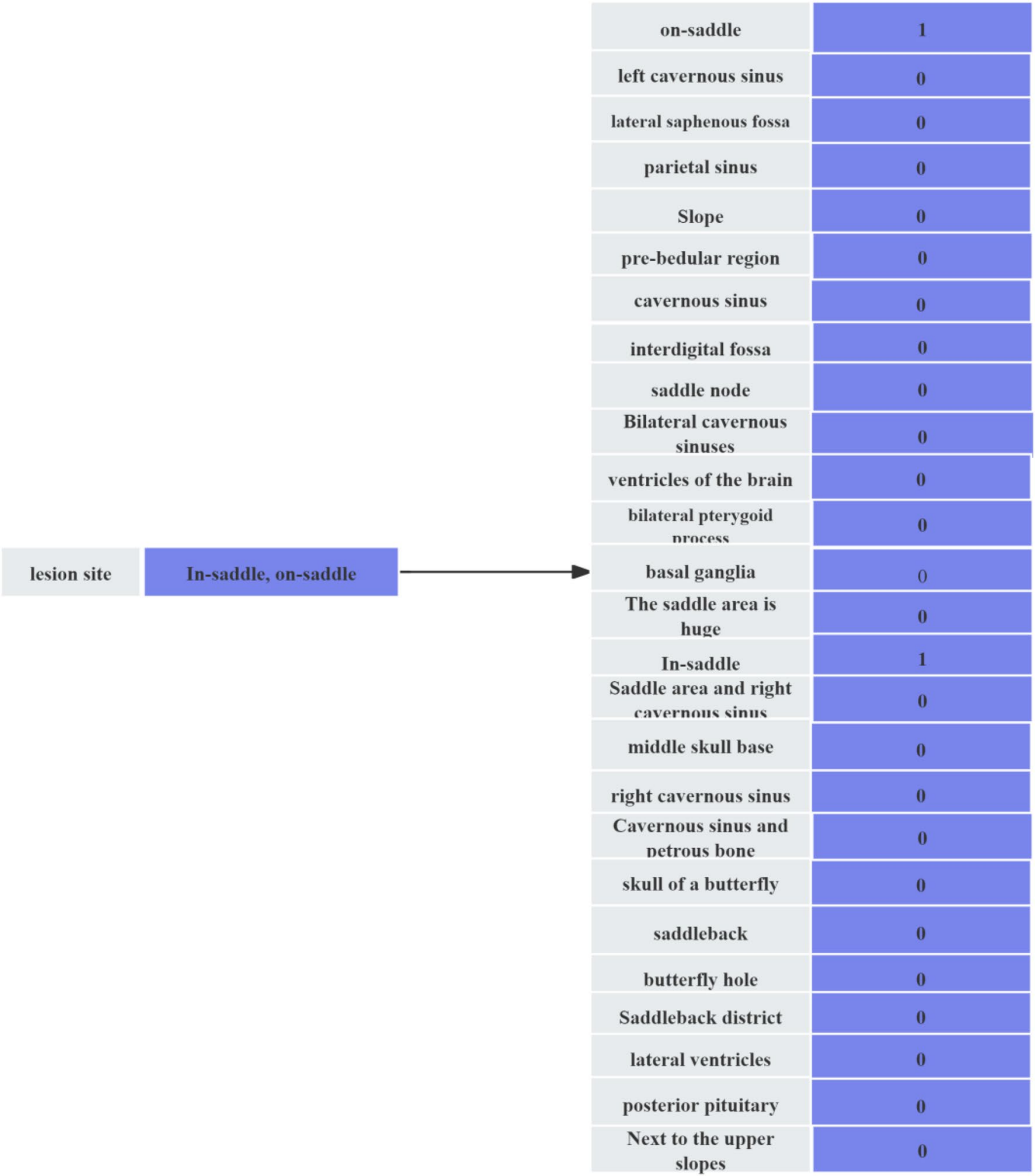
Deconstructed diagram of the lesion site.

The lesion site features 26 different sites, and the values of the original dataset are a combination of these 26 features. The lesion site is deconstructed by splitting all the sites it contains into dummy variables, and the schematic diagram of the deconstruction is shown in Fig. 1.

Up to this point, we have obtained two datasets. One of them is the dataset Data1 with serial number, name, age, lesion site, and pituitary tumor size excluded, and the dimensionality of the dataset is (158 × 15); the other dataset is the dataset Data2 with the addition of the age feature after binning and the deconstruction of the lesion site as a dummy variable after exclusion of serial number, name, age, lesion site, and pituitary tumor size, and the dimensionality of the dataset is (158 × 42).

#### Feature selection

This study added 26 new dimensions after structuring the lesion site in dataset Data2, and training the model using all the features may lead to a dimensionality catastrophe. In addition, the common features of datasets Data1 and Data2 may have noisy features that do not have any valid information for the prediction task. Therefore, in order to reduce the dimensionality of the dataset, reduce the computational requirements and improve the efficiency of the model as well as to reduce the interference of noise and irrelevant information, feature selection is performed on the datasets Data1 and Data2. In this study, no filter, Filter method and Wrapper method are adopted for data feature selection. Among them, Filter methods include three kinds: independence test (chi2Test), chi-square selection method (chi2Select) and mutual information selection method (mutual-Select); Wrapper methods also include three kinds: Lasso screening, GA screening, and RFECV screening, with a total of seven feature screening methods. The application of each method to each dataset (Data1 and Data2) produces one processed dataset, and since seven methods were used, a total of 14 processed datasets were obtained up to this point.

#### Model building

In this study, Python 3.9.10 and PyCharm tools were used to divide the dataset into a training set and test set in the 8–2 ratio based on the sci-kit-learn library, light gum library, and boost library. The training selected data is used to build and train the model, and the test set data is used to evaluate and validate the model. Modeling was performed on 14 processed datasets using eight machine learning algorithms; the eight machine learning algorithms include: BP neural network model, Logistic Regression model (LR), Decision Tree model (DT), Support Vector Machine model (SVM), Random Forest (RF) model, LightGBM model, XGBoost model and AdaBoost model. These eight machine learning algorithms were applied to seven feature selection methods on two different datasets, up to which 56 algorithmic models were obtained for each dataset.

#### Model evaluation

**Figure 2:**
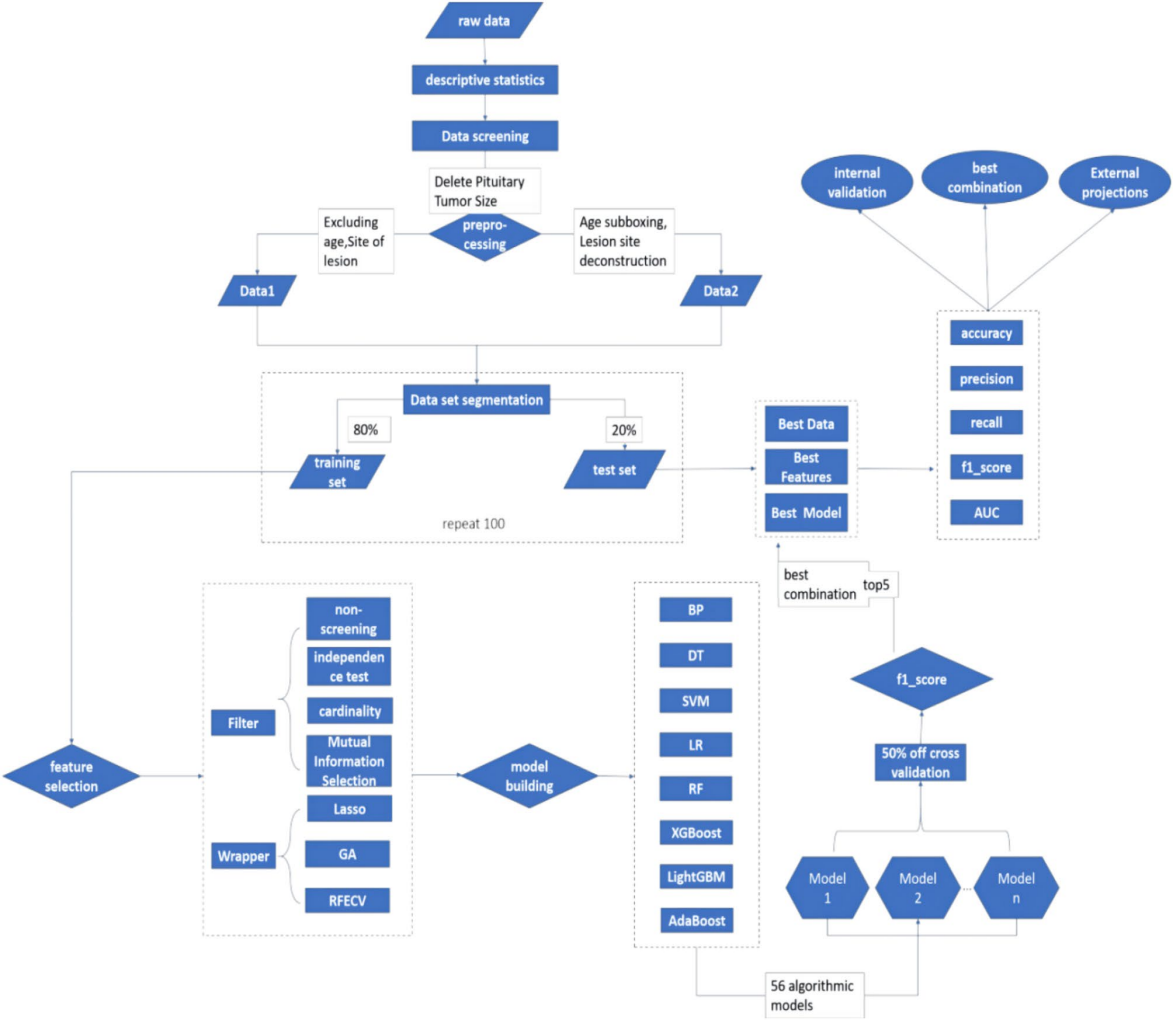
Machine learning-based early warning modeling process for the risk of olfactory impairment in patients undergoing transnasal pterygoid region pituitary tumor resection.

In this study, Python 3.9.10 and PyCharm tools were used to divide the dataset into a training set and test set in the 8–2 ratio based on the sci-kit-learn library, light gum library, and boost library. The training selected data is used to build and train the model, and the test set data is used to evaluate and validate the model. Modeling was performed on 14 processed datasets using eight machine learning algorithms; the eight machine learning algorithms include BP neural network model, Logistic Regression model (LR), Decision Tree model (DT), Support Vector Machine model (SVM), Random Forest (RF) model, LightGBM model, XGBoost model and AdaBoost model. These eight machine learning algorithms were applied to seven feature selection methods on two different datasets, up to which 56 algorithmic models were obtained for each dataset. See Fig. 2^9^.

### Statistical methods

Python 3.9.10 software was used for data analysis, count data were expressed by frequency and percentage, and comparisons were made by chi-square test; multifactorial logistic regression analysis was used to clarify the risk factors affecting olfaction, and the influencing factors with *P* < 0.05 were included in machine learning modeling, the area under the ROC curve (AUC) was plotted, and the analysis was performed to analyze the accuracy rate, precision rate, recall rate, and F1 value, and *P* < 0.05 was regarded as statistically significant difference (Supplementary Information).

## Results

### Essential characteristics of the investigation subjects

In this study, a total of 158 patients were collected from transnasal pterygoid region pituitary tumor resection, with a mean age of 54.7 ± 11.5 years, of which 83 cases (52.53%) were male, and the number of female patients was 75 cases (47.47%); There were 116 patients (73.42%) with postoperative olfactory impairment and 19 dimensions of explanatory variables and one dimension of explanatory variables was collected. The results of the univariate analysis showed that among the single influencing factors on whether patients with pituitary tumors suffered from olfactory dysfunction, the effects of five factors, namely, age, pituitary tumor stroke, blood crust formation, cerebrospinal fluid leakage, and length of surgery were statistically significant (*P* < 0.05) when the significance level a = 0.05 was achieved, as shown in Tables 3 and 4.

**Table 3:** Information related to pituitary tumor resection in the transnasal pterygoid region.

Factors	Quorum	Percent (%)
Age		
≤ 65	32	20.25
> 65	126	79.75
Sex		
Male	83	52.53
Female	75	47.47
Smoking history		
Yes	54	34.18
No	104	65.82
Drinking history		
Yes	67	42.41
No	91	57.59
Underlying disease		
Yes	54	34.18
No	104	65.82
Allergy history		
Yes	9	5.70
No	149	94.30
Pituitary tumor size		
Microadenoma	2	1.27
Macroadenoma	156	98.73
Cerebrospinal fluid leakage		
Yes	60	37.97
No	98	62.03
Pituitary tumor stroke		
Yes	24	15.19
No	134	84.81
Is the nasal pterygoid sinus infected		
Yes	8	5.06
No	150	94.94
Formation of bloody scabs		
Yes	101	63.92
No	57	36.08
Mucous membrane erosion		
Yes	5	3.16
No	153	96.84
Nasal mucus		
Yes	45	28.48
No	113	71.52
Sinus effusion		
Yes	15	9.49
No	143	90.51
Surgeries lasting more than 2 h		
Yes	64	40.51
No	94	59.49
Types of pituitary tumors		
Prolactin adenoma	14	8.86
Growth hormone tumor	17	10.76
Adrenocorticotropic hormone adenoma	16	10.13
Thyrotropinoma	4	2.53
Luteinizing hormone/follicle stimulating hormone tumors	18	11.39
Non-functioning pituitary tumor	89	56.33

**Table 4:** Univariate analysis of data related to pituitary tumor resection in the transnasal pterygoid region.

Factors	Olfactory impairment: Yes (n = 116)	Olfactory impairment: No (n = 42)	*χ*^2^	*P*
Age			5.0071	0.025
≤ 65	98	28		
> 65	18	14		
Sex			0.000	1.000
Male	61	22		
Female	55	20		
Smoking history			2.477	0.115
Yes	35	19		
No	81	23		
Drinking history			0.379	0.538
Yes	47	20		
No	69	22		
Underlying disease			0.000	1.000
Yes	40	14		
No	76	28		
Allergy history			0.007	0.933
Yes	6	3		
No	110	39		
Pituitary tumor size			0.0026	0.959
Microadenoma	1	1		
Macroadenoma	115	41		
Cerebrospinal fluid leakage			15.034	< 0.001
Yes	55	5		
No	61	37		
Pituitary tumor stroke			8.703	0.003
Yes	24	0		
No	92	42		
Is the nasal pterygoid sinus infected			1.785	0.181
Yes	8	0		
No	108	42		
Formation of bloody scabs			28.951	< 0.001
Yes	89	12		
No	27	30		
Mucous membrane erosion			0.727	0.394
Yes	5	0		
No	111	42		
Nasal mucus			1.908	0.167
Yes	37	8		
No	79	34		
Sinus effusion			2.335	0.127
Yes	14	1		
No	102	41		
Surgeries lasting more than 2 h			41.153	< 0.001
Yes	29	35		
No	87	7		
Types of pituitary tumors			4.995	0.416
Prolactin adenoma	8	6		
Growth hormone tumor	15	2		
Adrenocorticotropic hormone adenoma	12	4		
Thyrotropinoma	2	2		
Luteinizing hormone/follicle stimulating hormone tumors	13	5		
Non-functioning pituitary tumor	66	23		

### Cross-validation results on the training set

#### Comparative analysis of different data sets

**Figure 3:**
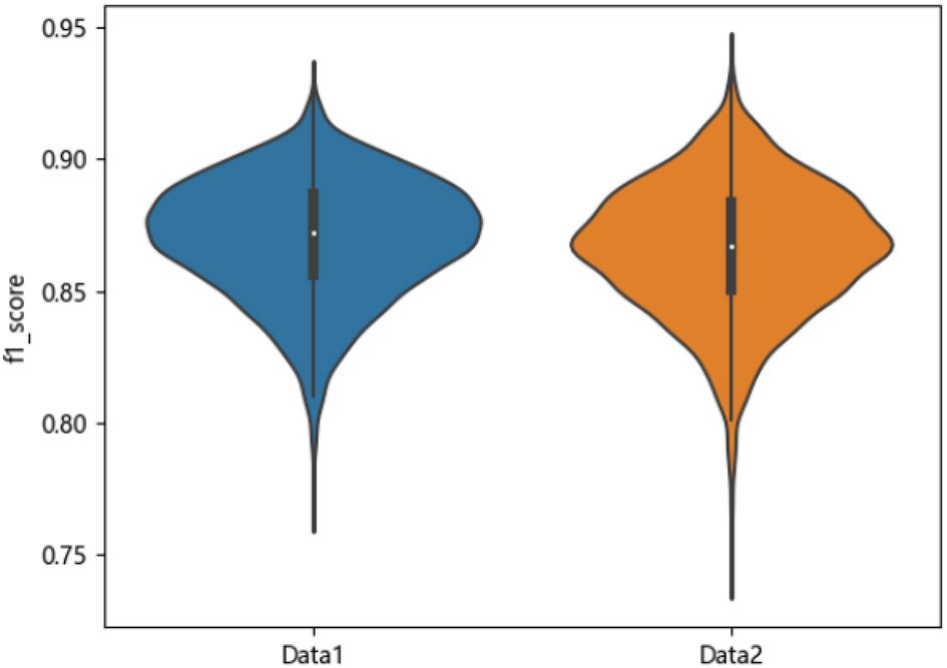
Box lines for the two datasets f1_score.

**Figure 4:**
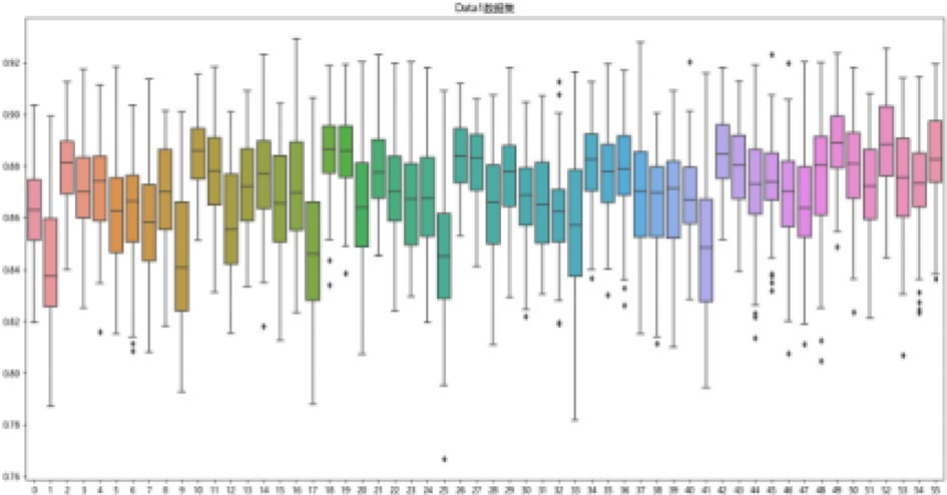
Boxplot representing cross-validation of the training set f1_score for 56 models on dataset Data1.

**Figure 5:**
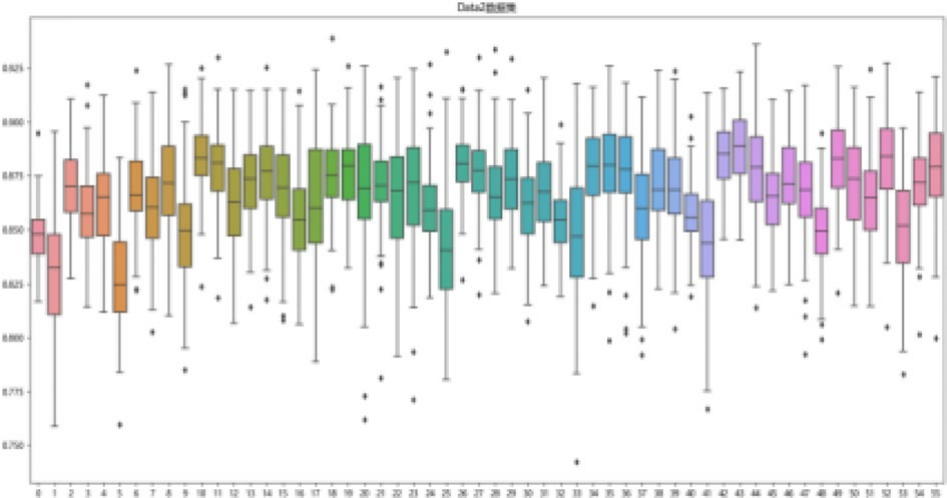
Boxplot representing cross-validation of the training set f1_score for 56 models on dataset Data2.

First, the degree of influence of different data processing methods of data on the risk prediction model of olfactory impairment in postoperative patients with pituitary tumors in the transsphenoidal region is considered. Taking f1_score as the evaluation index, box line diagrams are plotted as shown in Fig. 3 by counting the cross-validation results of 56 models on 100 times randomly divided training sets. The box line diagrams of 56 models on two data sets are shown in Figs. 4 and 5.

Figures 3, 4, and 5 are visible: dataset Data1 has a higher median and more concentrated data than Data2, which has a more dispersed data distribution. Although Data2 has a smaller minimum f1_score, its overall f1_score is higher and more pronounced in terms of high f1_score.

#### Comparative analysis of feature selection methods

The f1_score and standard deviation of the cross-validation results of no filtering, three Filter filtering methods, and three Wrapper filtering methods on the training set with 100 random divisions were computed on the datasets Data1 and Data2, respectively, and the results are shown in Table 5.

**Table 5:** Results of cross-validation of different feature selection methods.

Feature selection methods	Data1		Data2	
	Mean	Std	Mean	Std
All	0.864	0.023	0.853	0.026
chi2Test	0.868	0.023	0.870	0.024
chi2Select	0.871	0.023	0.868	0.024
Mutual-select	0.869	0.022	0.866	0.023
Lasso	0.870	0.022	0.867	0.025
GA	0.870	0.022	0.870	0.024
RFECV	0.879	0.020	0.869	0.024

Based on the results in Table 5, it can be seen that the f1_score is higher than the no feature selection method on both datasets using the 3 Filter and 3 Wrapper feature selection methods. Also, from the standard deviation point of view, the variance is higher when modeling with all features than when using the feature selection method. By performing a normality test (K–S test) and one-sample *t*-test on the mean values of f1_score for the six feature selection methods for the datasets Data1 and Data2, the results show that on both datasets, the f1_score of the feature selection methods is significantly better than the case of no feature selection (*P* < 0.05). Thus, the Filter feature and Wrapper selection methods improve the model's cross-validation on the training set. The results of the normality test and one-sample *t*-test are detailed in Table 6.

**Table 6:** K–S test and one-sample *t*-test p_value.

	K–S test	One-sample *t*-test
Data1	< 0.001	0.006
Data2	< 0.001	< 0.001

#### Evaluation results on test sets

**Figure 6:**
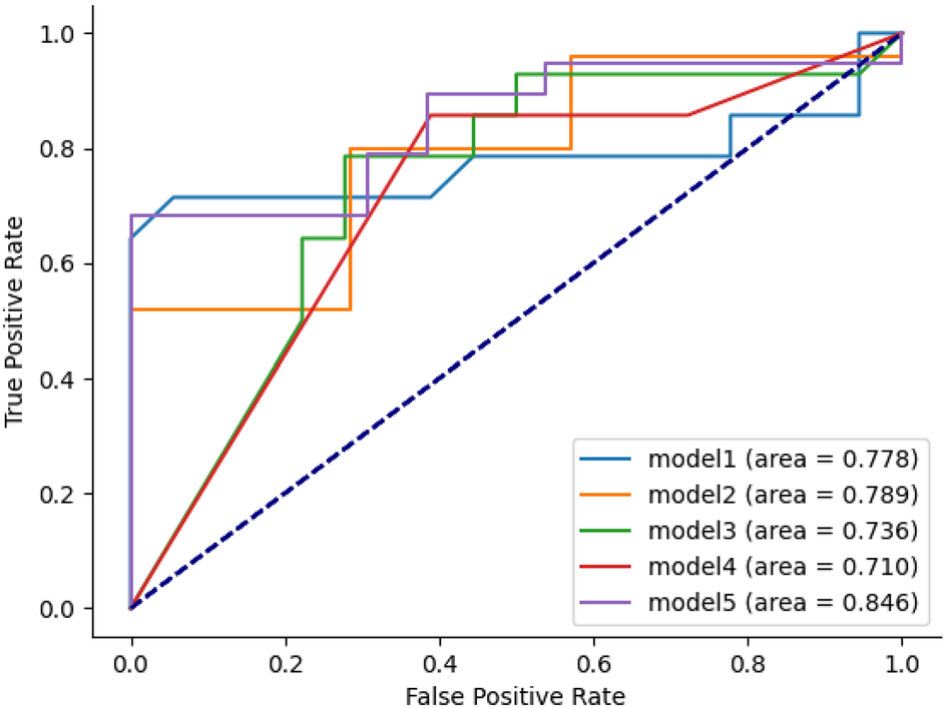
ROC curve.

**Figure 7:**
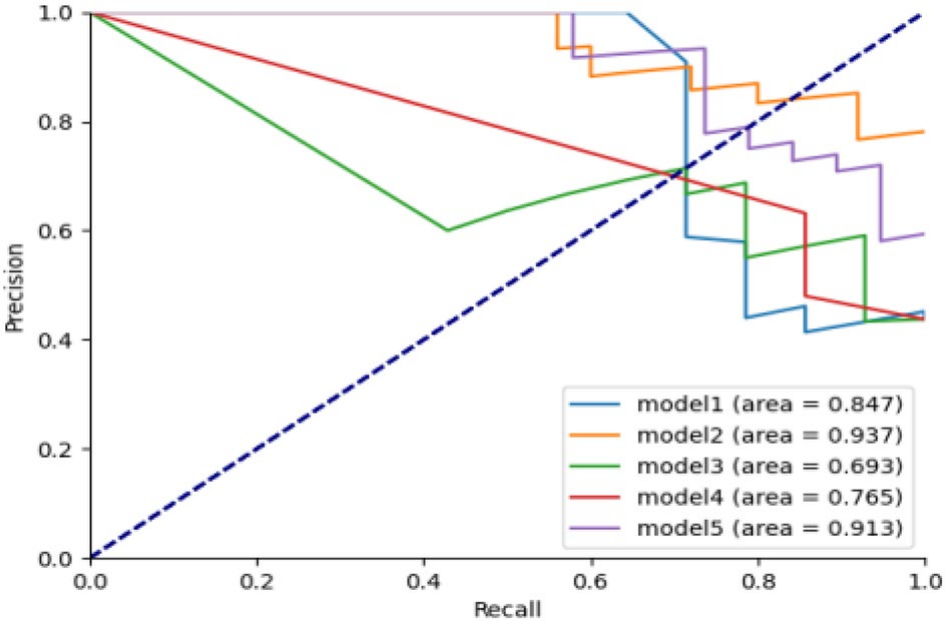
P–R curve.

In this study, we select the best-performing 5 combinations from the cross-validation results of 2 datasets, seven feature selection methods, and eight modeling methods on 100 randomly divided training sets. We test them externally on their corresponding test sets using the same feature selection and modeling methods. We comprehensively evaluate the performance of the models in terms of accuracy, precision, recall, F1 value, and AUC, and the results are shown in Table 7. The ROC plots and P-R plots are shown in Figs. 5 and 6. The top-to-bottom in Table 7 represents model 1- model 5 in Figs. 6 and 7, respectively.

**Table 7:** External tests of the five best models in cross-validation.

Model	Data set	Feature screening	Number of variables	Accuracy	Precision	Recall rate	F1 value	AUC
LR	Data2	chi2Select	6	0.500	0.462	0.857	0.600	0.778
RF	Data2	Lasso	8	0.750	0.870	0.800	0.833	0.789
RF	Data2	Mutual-Select	6	0.688	0.591	0.929	0.722	0.736
DT	Data2	Mutual-Select	6	0.688	0.600	0.857	0.706	0.710
RF	Data2	Lasso	9	0.750	0.720	0.947	0.818	0.846

As can be seen from Table 7, the highest values of the five evaluation metrics are found in the model that uses the Lasso method for feature selection, and the machine learning model is RF. Model 2 has the highest accuracy, precision, and F1 values, and model 5 has the highest accuracy, recall, and AUC. The highest accuracy, precision, recall, F1, and AUC values are 0.750, 0.870, 0.947, 0.833, and 0.846, respectively. Models 2 and 5 have 8 and 9 predictors selected by the Lasso method. The standard eight predictors were the length of surgery, gender, type of pituitary tumor, pituitary tumor stroke, nasal adhesions, age, cerebrospinal fluid leakage, and blood crust formation. Model 5 selected one more predictor, smoking history, compared to model 2. The results are shown in Table 8.

**Table 8:** Table of predictors for model 2 and model 5.

Model 2	Model 5
Duration of surgery	Duration of surgery
Sex	Smoking history
Types of pituitary tumors	Sex
Nasal mucus	Types of pituitary tumors
Age	Pituitary tumor stroke
Pituitary tumor stroke	Nasal mucus
Cerebrospinal fluid leakage	Age
Formation of bloody scabs	Cerebrospinal fluid leakage formation of bloody scabs

In summary, among the established machine learning models, the random forest model has the highest AUC of 0.846, and the accuracy, precision, recall, and F1 values of 0.750, 0.870, 0.947, and 0.833 demonstrate the optimal prediction efficacy, respectively.

## Discussion

Among the surgical approaches for the treatment of pituitary tumors, transsphenoidal pituitary tumor resection resides as the conventional choice. Despite its widespread adoption, the procedure is associated with various potential complications, including olfactory impairment, the latter of which has become a significant clinical challenge^11^. Impaired olfactory function is not only capable of triggering loss of appetite in patients but also poses a threat to patient safety and may lead to anxiety, depression, and changes in behavioral and cognitive functioning^12^. The specific pathogenesis of olfactory dysfunction has yet to be clearly explained. In addition, there are challenges in the early detection and treatment of olfactory dysfunction. This shortcoming is particularly pronounced when traditional statistical analysis and modeling methods are employed. In light of this, machine learning algorithms, by deeply mining and analyzing the underlying variables and internal informative relationships in the data, have the potential to enhance the model generalization capabilities, thus providing new perspectives and solutions for the prediction of such complications^13^.

A total of 158 patients who underwent transnasal pterygoid region pituitary tumor resection were included in this study: 116 patients (73.42%) with postoperative olfactory dysfunction and 42 patients (26.58%) without olfactory dysfunction. The dataset has features such as high dimensionality and noise, and using highly unbalanced features to train the model may result in a catastrophe of dimensionality. Therefore, to reduce the dimensionality of the dataset, reduce computational requirements, improve the model's efficiency, and reduce the interference of noise and irrelevant information. In this study, seven feature selection methods, namely, no screening, independence test (chi2Test), chi-square selection (chi2Select), mutual information selection (mutual-Select), Lasso screening, GA screening, and RFECV screening, are used for feature selection to explore and optimize the dataset-based dimensionality reduction strategy^14^. Among them, Lasso screening is widely used for variable screening of complex data. Lasso screening reduces the false positive rate by reducing multicollinearity, improving the model prediction effectiveness, and reducing the prediction consumption^15^.

In this study, critical indicators, including length of surgery, type of pituitary tumor, pituitary tumor stroke, nasal adhesions, cerebrospinal fluid leakage, blood crust formation, age, gender, and smoking history were identified as potentially crucial in developing a predictive model for postoperative olfactory impairment. Consistent with the findings of Martin et al.^6^, there was a correlation between the presence of olfactory dysfunction and several critical indicators, particularly length of surgery and nasal adhesions. The development of postoperative olfactory impairment is thought to be closely related to damage to the olfactory anatomy, with olfactory cells located mainly in the mucosa of the upper turbinate and nasal septum. Due to the unique surgical access of pituitary tumor resection in the transnasal pterygoid region, it inevitably causes tearing and prolonged compression of the nasal mucosa, which in turn leads to olfactory dysfunction^16^. Hsu et al.^17^ investigators have also found that widening the surgical access scope increases the defect's size at the skull's base, leading to a higher risk of cerebrospinal fluid leakage and impairment of olfactory function. Age is also a known risk factor for olfactory impairment; for example, Braun et al.^18^ found that myelin fiber density is lower in older people compared to younger people, which can reduce olfactory sensitivity. Long-term smoking can damage the defense ability of the nasal mucosa, and intraoperative mechanical stimulation and increased inflammatory response can cause edema of the nasal mucosa, which affects olfactory pigment conduction and increases the risk of postoperative olfactory impairment^19^. Therefore, when performing transnasal pterygoid region pituitary tumor resection, it is crucial to control the duration of the operation, reduce the damage to the nasal mucosa, and perform skull base reconstruction to avoid cerebrospinal fluid leakage. Using machine learning algorithms, this study constructed different predictive models and analyzed the optimal subset of features of the models to identify significant predictors of postoperative olfactory impairment. While the insights these models provide are robust, their specific biological mechanisms require further in-depth study to understand better and prevent the development of postoperative olfactory impairment.

As a field of artificial intelligence, machine learning algorithms can automatically learn from inputs or data experiences and convert these data into specialized skills or knowledge to assist clinicians in making more accurate clinical decisions based on patient indicators^20^. In this study, a variety of performance evaluation metrics such as accuracy, precision, recall, F1 score, and area under the subject operating characteristic (ROC) curve (AUC) were comprehensively used to assess the efficacy of the constructed model through a detailed data preprocessing process, including steps such as data cleansing, filtering, filling in missing values, feature selection, and model construction. In this study, 112 different models were built with the help of eight machine-learning algorithms: BP, LR, DT, SVM, RF, LightGBM, XGBoost, and AdaBoost. A comprehensive analysis of five key evaluation metrics showed that the Random Forest (RF) model performed the best. In terms of predicting postoperative olfactory impairment after transsphenoidal pituitary tumor surgery, its maximum values of accuracy, precision, recall, F1 score, and AUC reached 0.750, 0.870, 0.947, 0.833, and 0.846, respectively, which coincided with the results of the study by Jianchang Hu et al.^21^. Random forest is a homogeneous and integrated learning method that can construct multiple base learners and pool their predictive power to enhance the overall performance^22^. It searches for an optimal subset of features through an ordered combination of the training set and features. It improves the general prediction of the model by increasing its importance in the decision-making process^23^. Therefore, the random forest model has potential clinical applications in predicting olfactory impairment in postoperative patients with pituitary tumors.

Innovations of this study: (1) In China, relatively few studies have been conducted on the risk warning model for olfactory impairment in postoperative pituitary tumor patients, and the machine learning model successfully built in this study will provide clinicians with an innovative and highly accurate prediction tool, which will help them to formulate a more effective treatment strategy, and thus potentially improve the clinical prognosis of patients. (2) Compared to the status quo, where only a single prediction model is commonly constructed, this study used eight different machine learning models and model construction through 112 different algorithms, which were cross-validated with five folds and comprehensively evaluated across 1400 models. Eventually, the overall assessment of model performance is based on multi-dimensional evaluation indexes such as accuracy, precision, recall, F1 score, and AUC, which ensures the accuracy and reliability of the model prediction and selects the best prediction model, significantly improving the model's practical value.

Shortcomings of this study: (1) The data samples in this study are limited, and the lack of sufficient data volume and representative samples may affect the stability and generalization ability of the model due to the limitation of specific surgeries, the small range of cases collected during the period of the subject study at the stage of spreading of the epidemic, and the morbidity rate of the patients; therefore, further multicenter and large-sample related studies need to be carried out in the follow-up. (2) Although the model may have specific predictive performance, its applicability in actual clinical applications still requires further research and validation. The acceptance of the prediction results by doctors and patients, the combination of the model with clinical experience, and the corresponding interventions need to be studied in depth.

In conclusion, the Random Forest prediction model has a good effect on predicting olfactory dysfunction in postoperative patients with transsphenoidal pituitary tumors. The length of surgery, gender, type of pituitary tumor, pituitary tumor stroke, nasal adhesion, age, cerebrospinal fluid leakage, scab formation, and history of smoking are the critical risk factors for the occurrence of olfactory dysfunction in postoperative patients. With the help of an olfactory dysfunction risk prediction model, the prediction model can inform medical practitioners to develop individualized treatment strategies and proactive interventions to reduce the risk of olfactory dysfunction based on patients' personal characteristics and clinical data. Further, through the in-depth mining and analysis of large clinical data sets, the potential factors and biological mechanisms closely related to olfactory impairment can be revealed, laying the foundation for subsequent research and providing more solid scientific support for the prevention and treatment of olfactory impairment.

### Supplementary Information

Supplementary Information.

## Data Availability

The dataset generated and analyzed during the current study is available from the corresponding authors on reasonable request.
